# The Contribution of Bilingualism, Parental Education, and School Characteristics to Performance on the Clinical Evaluation of Language Fundamentals: Fourth Edition, Swedish

**DOI:** 10.3389/fpsyg.2019.01586

**Published:** 2019-07-17

**Authors:** Ketty Andersson, Kristina Hansson, Ida Rosqvist, Viveka Lyberg Åhlander, Birgitta Sahlén, Olof Sandgren

**Affiliations:** ^1^ Department of Clinical Sciences Lund, Logopedics, Phoniatrics, and Audiology, Faculty of Medicine, Lund University, Lund, Sweden; ^2^ Department of School Development and Leadership, Faculty of Education and Society, Malmö University, Malmö, Sweden

**Keywords:** language assessment, bilingualism, academic achievement, language exposure, language disorder

## Abstract

Assessment of bilingual children in only one language fails to acknowledge their distributed linguistic competence and has been shown to overidentify language disorder in bilingual populations. However, other factors, sometimes associated with bilingualism, may also contribute to low results in language assessments. Our aim was to examine the impact of these factors on language abilities. We used the Clinical Evaluation of Language Fundamentals – Fourth Edition, Swedish (CELF-4) to investigate core language abilities of 224 7- to 8-year-old children. Results showed 30 and 80% of monolinguals and bilinguals, respectively, performing more than 1 SD below the normative sample mean, calling into question the clinical utility of the test. However, participant and school characteristics provided a deeper understanding of the skewed results. In isolation, bilingualism predicted 38% of the variance in the CELF-4 Core scores. With level of parental education entered the variance explained by the model increased to 52%, but the unique contribution of bilingualism was reduced to 20%. Finally, with information added on school characteristics and enrollment in the school’s recreation center the model explained an additional two percent, with the unique contribution of bilingualism further reduced to 9%. The results indicate an increased risk for low results on the CELF-4 Core when children present with multiple risk factors. This highlights the need to look beyond bilingualism in language assessment of bilingual children and adolescents and to consider other explanations to academic struggle. Available interventions must be considered and applied proportionately to their respective impact on the individual’s development.

## Introduction

Large-scale international comparisons have reported a decline in Swedish primary and elementary school students’ academic attainment the last 20 years, as compared to peers in other countries. Swedish 15-year olds’ skills and knowledge in reading, mathematics, and science, as assessed in the Program for International Student Assessment (PISA), have steadily declined from 2000. The scores reached an all-time low in 2012 (OECD, 2014) and returned to the OECD mean in 2015 (OECD, 2016). Similar developmental trends have been shown for fourth grade reading comprehension [Progress in International Reading Literacy Study (PIRLS 2016)] and fourth and eighth grade knowledge in mathematics and science [Trends in International Mathematics and Science Study (TIMSS 2015)].

The most recent report from the OECD indicates that the negative trend among Swedish students may be reversed, or at least halted (OECD, 2016). However, the results have caused great concern in the general public. Some policy makers have used the ensuing discussion to make ideologically based claims on necessary changes to the school curriculum.

Research-based analyses have offered several explanations to the declining results. Some question the validity (Brunner et al., 2007) and reliability (Goldstein, 2004) of the large-scale assessments of student performance used in international comparisons. Declining results have been linked to factors such as less able teachers (Meroni et al., 2015), low teacher expectations on student progress (Wang et al., 2018), increased use of computers and handheld devices in classrooms (OECD, 2015), and lack of large-scale funding and coordination of systematic evaluations of educational practices (Pontoppidan et al., 2018). In a Swedish context, high levels of autonomy for school districts have led to greater differences between schools (Swedish National Agency for Education, 2006). Globally, rapid changes in the student cohort demographics, from largely monolingual to bilingual, have been presented as a main (Agirdag and Vanlaar, 2018) or a contributing (OECD, 2018b) factor.

Indeed, many Western countries have seen an increased number of bilinguals (OECD, 2018a), and language is of crucial importance for school success (Pace et al., 2019). Mainstream teaching requires students to be fluent in the majority language not only to follow the teacher’s instructions and to participate in the teaching activities but also to access the hidden curriculum, which guides school culture (Baker, 2011). Language, in particular vocabulary and listening comprehension, has repeatedly been shown to be at the core of these competencies. The vocabulary used in classrooms differs greatly from that used in everyday conversation, with more abstract words (Cummins, 1979), and bilingual children need support to keep pace with monolingual peers linguistically and academically. Although bilingual children tend to develop their vocabulary knowledge at the same pace as monolingual children, the gap between the groups remains because bilingual children have a lower starting point and monolinguals gradually improve and, thereby, constitute a continuously moving target (Thordardottir and Juliusdottir, 2013). In order to make similar academic and language achievements as monolinguals, a high oral language proficiency is required (Babayiğit, 2015). Indeed, O’Connor et al. (2018) found no difference between mono- and bilingual children regarding literacy and numeracy when the bilingual children had a high proficiency in receptive English vocabulary. However, bilingual children with less developed receptive vocabulary skills had difficulties meeting the school demands.

However, several factors have impact on language development, only one of which is bilingualism. Children, whether Monolingual and bilingual children alike, living in less affluent areas risk facing “a double dose of disadvantage,” experiencing impoverished language input at both home and school (Neuman et al., 2018, see also Hoff, 2013). Neuman et al. (2018) found parents in socioeconomically disadvantaged areas to use fewer words and shorter, less complex sentences when interacting with their children, than parents in a more diverse working-class comparison neighborhood. In addition, Neuman et al. (2018) found similar differences in conversation in school; teachers in the less affluent neighborhood used less varied vocabulary and less complex syntax than the teachers in the working-class neighborhood, thereby failing to provide compensation for the limited language input in these children’s home environments. With a reduced experience of interaction with adults who use complex syntax and vocabulary, known to enhance children’s expressive language development, these children start school with less robust language experiences, which, in turn, increase the risk of school failure (Neuman et al., 2018).

Agirdag and Vanlaar (2018) criticize dichotomous categories of bilingualism, as used by, for example, OECD, and point to the need for evaluation of language exposure and use, as more reliable predictors of academic outcomes. The authors compared two competing views on bilingualism; the time-on-task perspective, which predicts a monolingual advantage in outcome; and the additive perspective on multilingualism, which predicts that transfer and switching between languages will have positive cognitive and linguistic effects and hence a bilingual advantage. Agirdag and Vanlaar (2018) failed to show a bilingual advantage. Bilinguals showed lower achievements in reading and mathematics than monolingual peers. Taking school and student characteristics into account reduced the achievement gap between monolingual and bilingual children, but not to an insignificant level. However, an in-depth analysis of the language exposure, taking into account which language the child used in different conversational contexts, provided more information. Bilingual children who regularly used the home language with their parents achieved in level with monolingual peers. In addition, speaking the majority language with friends was positively associated with academic achievements (Agirdag and Vanlaar, 2018). Similarly, Huang et al. (2018) found that using the second language in the spare time was more influential on language comprehension than using the language in the classroom. Thus, bilingual children who receive high-quality input in their mother tongue and who can use their school language for everyday conversation with friends are likely to perform at the same level as monolingual peers. In fact, Agirdag and Vanlaar (2018) were able to show that these children may even outperform monolingual peers in societies with a positive view on bilingualism.

The use of monolingual language norms and expectations may lead to an overidentification of language problems in bilingual populations (Lugo-Neris et al., 2015). Children who acquire a second language can sometimes be hard to distinguish from children with developmental language disorder (DLD), with both groups presenting with similar language profiles, at least at some point in development (Salameh et al., 1996; Windsor and Kohnert, 2004). The importance of assessing both languages has long been stressed, but even when assessed in both languages, or in their strongest language, bilingual children from disadvantaged socioeconomic backgrounds are overidentified as having DLD (Barragan et al., 2018). In a sample of Spanish-English dual language learners, Barragan et al. (2018) found more than 50% to perform more than 1 SD below the mean on the Spanish version of the Clinical Evaluation of Language Fundamentals – Fourth Edition (CELF-4), that is, exceeding the recommended cut-off score for language disorder. The older children were more likely to show low performance on the expressive subtests (Recalling Sentences and Formulated Sentences) than the younger children, indicating a shift of language dominance at this age (Kohnert and Bates, 2002; Barragan et al., 2018). Norm-referenced tests risk overidentifying children from low-socioeconomic backgrounds, and separate norms may be necessary to improve the sensitivity and specificity of language assessments.

To sum up, bilingualism *per se* is not detrimental to children’s language outcomes and academic achievements. However, a number of factors, associated with increased risk of academic underachievement, may accumulate in bilingual children. We aim to disentangle the relative contributions of bilingualism, socio-economic disadvantage, and suboptimal language exposure and use on core language abilities.

## Purpose

The purpose of this study was to estimate the impact of bilingualism on CELF-4 Core scores in isolation and in combination with information on level of parental education, school characteristics (proportion of parents with tertiary education and proportion of students with Swedish as second language), and recreation center enrollment. We answer two questions:

1. How do monolingual and bilingual children perform on the Swedish CELF-4 Core?
2. How much unique and combined variance in CELF-4 Core scores can be attributed to bilingualism, level of parental education, school characteristics, and enrollment in the school’s recreation center?

## Materials and Methods

### Participants

CELF-4 Core scores were collected from 224 7- to 8-year-old children (*M*_age_ 90.8, SD 7.3, range 77–105 months), representing 57% of the students in first and second grade in six invited public schools from two school districts. The participants received education in Swedish with the exception of a weekly lesson of first language teaching for bilingual children, if requested by the parents (on a national level requested for 60% of eligible children; Swedish National Agency for Education, 2019). No preselection of participants was made on the basis of language risk or special education needs. The sample was representative of the student cohort regarding the proportion of mono- and bilingual participants [*t*(223) = 1.58, *p* = 0.12]. The distribution of boys and girls (120 girls and 104 boys) did not differ significantly [*χ*^2^(1) = 1.14, *p* = 0.29].

The parents of all participants provided information on level of parental education, children’s bilingualism status, and children’s enrollment in the school’s recreation center activities after school hours. Additional school characteristics (proportion of parents with tertiary education and proportion of students with Swedish as second language) were compiled from publicly available statistical data (Swedish National Agency for Education, 2019).

### Assessment

Participating children were assessed with the Swedish version of the CELF-4 (Semel et al., 2013). Four subtests compose a core language score used as a screening in clinical decision-making. The subtest Concepts and Following Directions requires the child to point to pictures following increasingly complex oral instructions from the examiner. Word Structure assesses morphological ability in a sentence completion format, where the child is required to mark noun, verb, and adjective inflections. In Recalling Sentences, the task is to give a verbatim repetition of a sentence, without modifications. In Formulated Sentences, the child freely formulates a sentence appropriate to a picture stimulus, including a target word provided by the examiner.

### Procedure

The study was carried out in accordance with the recommendations of the Ethics Review Board of Southern Sweden (approval number 2016/567) with written informed consent from the parents of all participants, in accordance with the Declaration of Helsinki. The teachers in participating schools and classrooms distributed parent consent forms. Parents who approved their child’s participation filled out a form with information on language exposure and use, level of parental education, previous speech-language pathology (SLP) or special education services provided for the child, and enrollment in the school’s recreation center activities after school hours. All examiners were native Swedish-speaking SLPs or final year SLP students specially trained for the purpose of the data collection. All testing was conducted during school hours in rooms adjacent to the child’s classroom. The testing took approximately 40 min. The subtests were administered in a fixed sequence, and all verbal instructions were scripted, in order to reduce the risk of inter- and intra-rater inconsistencies.

### Statistical Analyses

In accordance with the test manual, the raw scores from the subtests were converted to subscale scores with a mean of 10 and a SD of 3. The subscale scores were collapsed to form a core language score with a mean of 100 and a SD of 15, to allow comparison with the normative sample of the CELF-4.

From the sample of 224 participants, complete data on bilingualism status, level of parental education, and enrollment in the school’s recreation center were obtained for 170 participants (see Table 1). CELF-4 Core scores were obtained for 222 participants, with two children failing to participate in one of the CELF-4 subtests.

**Table 1:** Participants’ demographic information.

Demographics	*n*		
School district	A	B	Total
**Grade**			
First	52	48	100
Second	68	56	124
Total	120	104	224
**Level of parental education**			
≤9 years (elementary school)	22	2	24
12 years (high school)	42	6	48
>12 years (university)	34	92	126
Missing	22	4	26
**Bilingual**			
Yes	87	19	106
No	33	85	118
Missing	0	0	0
**Recreation center enrollment**			
Yes	50	97	147
No	34	6	40
Missing	36	1	37
**School characteristics**			
Parents with tertiary education (%)	45^a^ (56)	80^b^ (57)	
Students with Swedish as second language (%)	43^a^ (24)	25^b^ (25)	

*Missing = information missing in parental report*.

a *School year 2017–2018. National averages in parentheses*.

b *School year 2018–2019. National averages in parentheses*.

Publicly available data on the proportion of parents with tertiary education and proportion of students with Swedish as first language in the participating schools were ranked from lowest (1) to highest (6). The rank scores were summed to form an index of school characteristics (possible range 2–12). The highest index score was assigned to the school with highest proportion of parents with tertiary education and students with Swedish as first language.

Hierarchical regression was used to investigate the effect of the independent variables on the CELF-4 Core scores. Bilingualism was entered first into the model. In a second step, level of parental education was added to calculate the effect above and beyond that of bilingualism. In a final step, the index of school characteristics and enrollment in the school’s recreation center were added to the regression model. Preliminary analyses ensured all assumptions of normality, linearity, multicollinearity, and homoscedasticity were met. All statistical analyses were performed using SPSS version 25 for Windows.

## Results

On the CELF-4 Core, the mean score for the sample was 77.99 (SD = 23.93, range 40–122), which is almost 1.5 SDs below the normative sample of the test. On a group level, monolingual participants (*n* = 118) performed within the expected range (*M* = 91.81, SD = 16.8, range 49–122), whereas the bilingual participants (*n* = 104) performed below the normative range (*M* = 62.31, SD = 20.99, range 40–114). Although the participants, on an individual level, were not preselected on the basis of language or academic risk, 30% of monolingual participants, and 80% bilingual participants, performed more than 1 SD below the mean (≤85) on the CELF-4 Core index, the recommended cut-off score for language disorder.

Table 2 shows correlations between the dependent variable (CELF-4 Core) and the independent variables (Bilingualism, Level of parental education, School characteristics, Recreation center enrollment). All correlations were significant, indicating associations between the variables. To further explore the unique and shared variance in CELF-4 Core scores explained by the independent variables, all variables were entered into a hierarchical regression model (see Table 3). In the first model, bilingualism was entered as a single predictor, accounting for 38% of the variance in CELF-4 Core scores, *F*(1, 171) = 104.96, *p* < 0.001. In Model 2, level of parental education was added, increasing the proportion of explained variance to 52%, *F*(2, 170) = 91.22, *p* < 0.001. The unique variance explained by bilingualism decreased to 20%, while level of parental education explained 11.5% unique variance. Thus, the shared variance of bilingualism and level of parental education, as expected from the correlations in Table 2, are greater than or equal to the unique contribution. In the final model, school characteristics and enrollment in the school’s recreation center were entered, explaining an additional 2% of the variance in CELF-4 Core scores, *F*(4, 168) = 49.10, *p* < 0.001. Again, the unique contribution of bilingualism decreased, to 9%, an indication of the overlapping multifactorial influence of the independent variables.

**Table 2:** Correlations between CELF-4 Core, bilingualism, level of parental education, school characteristics, and enrollment in the school’s recreation center.

	1	2	3	4
1. CELF-4 Core				
2. Bilingualism	−0.62^***^			
3. Level of parental education	0.56^***^	−0.35^***^		
4. School characteristics	0.59^***^	−0.57^***^	0.53^***^	
5. Recreation center enrollment	0.41^***^	−0.44^***^	0.37^***^	0.41^***^

*** *p < 0.001*.

**Table 3:** Hierarchical regression model predicting CELF-4 Core scores.

	Model 1			Model 2			Model 3		
	*R*^2^ = 0.38			*R*^2^ = 0.52			*R*^2^ = 0.54		
				∆*R*^2^ = 0.14			∆*R*^2^ = 0.02		
	*B*	SEB	*β*	*B*	SEB	*β*	*B*	SEB	*β*
**Measure**									
Bilingualism	−29.49	2.88	−0.62^***^	−22.97	2.71	−0.48^***^	−18.30	3.17	−0.38^***^
Level of parental education				13.43	1.93	0.40^***^	10.71	2.14	0.32^***^
School characteristics							1.68	0.66	0.18^***^
Recreation center enrollment							2.80	3.53	0.05

*** *p < 0.001*.

## Discussion

For Swedish speech-language pathologists, the CELF-4 represents one of few norm-referenced standardized language assessments. Consequently, clinicians rely heavily on the results from CELF-4 assessments when making diagnostic decisions. The purpose of this study was to examine how monolingual and bilingual participants perform on the CELF-4 Core. We report unexpectedly low results, with 30% of monolingual participants scoring below the recommended screening cut-off score for language disorder, according to the test manual (Semel et al., 2013), despite similar prerequisites as the normative sample. The relative size of the samples may be one possible explanation. The study sample is greater than the CELF-4 normative sample for 7- to 8-year olds. The results of the bilingual participants, who were excluded from normative sample, were less surprising, but equally alarming, with 80% scoring below the cut-off score for language disorder.

The background variables offer a deeper understanding of the results. Bilingualism, explaining 38% of the variance in CELF-4 Core scores when analyzed separately, loses most of its predictive force when taking socioeconomic and school factors into account. The hierarchical regression model reveals high levels of shared variance between bilingualism, level of parental education, school characteristics, and enrollment in the school’s recreation center. For children who exhibit more than one risk factor, the effect is detrimental. Participants who speak Swedish as a second language, come from socioeconomically challenged home environments and who attend schools where many students share these circumstances are at an increased risk of low results on the CELF-4 Core, and, as a consequence, of being misidentified as having a language disorder. Although language support is required regardless the cause, children who experience suboptimal language learning conditions are likely to gain more from focused instruction on vocabulary and reading comprehension (Spencer et al., 2017). Individuals with language disorder, and the people around them, will also need to be equipped with compensatory strategies in order to be able to make necessary everyday adjustments to prevent the risk of language and communication breakdowns (Ebbels et al., 2019).

When analyzed in combination with socioeconomic and school factors, bilingualism only explained 9% of the CELF-4 Core scores. Consequently, separate norms for bilingual children or children from different socioeconomic circumstances would not provide a satisfactory solution nor would norm-referenced assessment in the first language. This would, in most cases, mean that the bilingual child once again is compared with a monolingual normative sample (for a discussion, see Scheidnes and Tuller, 2016). Instead, other types of language assessments should be more generally practiced. Dynamic assessment, focusing on the potential for language learning rather than providing a static assessment at one point in time, is one example (Hasson et al., 2013; Dockrel et al., 2015). Processing measures of language proficiency, for example, non-word repetition, have also shown higher sensitivity and specificity in bilingual populations than traditional language assessment (Thordardottir and Brandeker, 2013).

What, then, would increase the diagnostic accuracy in assessments of bilingual children? First, all assessments must take into account available demographic information, for example, level of parental education. Similar to the results presented here, Barragan et al. (2018) found more than 50% of children from low-income, bilingual backgrounds to perform in level with children with language disorder on CELF-4 assessments. We show the same applies to a high proportion of monolingual children with the same background.

Second, language assessments must evaluate bilingual children’s opportunities to use their second language in different contexts and with different conversational partners. Agirdag and Vanlaar (2018) demonstrate the positive effect on academic results of speaking the second language with schoolmates and friends. Information on second language use with peers outside school hours, although not contributing significantly to the model as measured with enrollment in the school’s recreation center, should be further investigated.

Third, schools are required to face the challenge of providing equitable education services to students of different language and socioeconomic backgrounds to make the curriculum content accessible for all. This calls for school environments with clearly defined areas within the classroom for different teaching activities, and high-quality teaching methods, for example, interactive book reading, structured conversations and targeted feedback, in order for the school hours to be used optimally (Dockrell et al., 2015). With these measures, all students will have a better chance of performing to their capacity within classrooms that accept and invite all voices and languages of the students to be heard (Rolstad et al., 2005).

## Data Availability

The raw data supporting the conclusions of this manuscript will be made available by the authors, without undue reservation, to any qualified researcher.

## Ethics Statement

This study was carried out in accordance with the recommendations of the Ethics Review Board of Southern Sweden (approval number 2016/8) with written informed consent from all subjects. All subjects gave written informed consent in accordance with the Declaration of Helsinki.

## Author Contributions

KA, KH, VL, BS, and OS were responsible for the concept and design of the study. KA, KH, IR, and OS collected the data. KA performed statistical analyses and interpreted the data. OS wrote the first version of the manuscript. All authors critically revised the manuscript and approved the submitted version.

### Conflict of Interest Statement

The authors declare that the research was conducted in the absence of any commercial or financial relationships that could be construed as a potential conflict of interest.

## References

[ref1] Agirdag O. Vanlaar G. (2018). Does more exposure to the language of instruction lead to higher academic achievement? A cross-national examination. Int. J. Biling. 22, 123–137. 10.1177/1367006916658711

[ref2] Babayiğit S. (2015). The relations between word reading, oral language, and reading comprehension in children who speak English as a first (L1) and second language (L2): a multigroup structural analysis. Read. Writ. 28, 527–544. 10.1007/s11145-014-9536-x

[ref3] Baker C. (2011). Foundations of bilingual education and bilingualism. 5th Edn. Bristol, UK; Tonawanda, NY: Multilingual Matters.

[ref4] Barragan B. Castilla-Earls A. Martinez-Nieto L. Restrepo M. A. Gray S. (2018). Performance of low-income dual language learners attending English-only schools on the clinical evaluation of language fundamentals – Fourth edition, Spanish. Lang. Speech Hear. Serv. Sch. 49, 292–305. 10.1044/2017_LSHSS-17-0013 29330555 PMC5963037

[ref5] Brunner M. Artelt C. Krauss S. Baumert J. (2007). Coaching for the PISA test. Learn. Instr. 17, 111–122. 10.1016/j.learninstruc.2007.01.002

[ref6] Cummins J. (1979). Cognitive/academic language proficiency, linguistic interdependence, the optimum age question and some other matters. Work. Papers Bilingual. 19, 1–9.

[ref7] Dockrell J. E. Bakopoulou I. Law J. Spencer S. Lindsay G. (2015). Capturing communication supporting classrooms: The development of a tool and feasibility study. Child Lang. Teach. Ther. 31, 271–286. 10.1177/0265659015572165

[ref8] Ebbels S. H. McCartney E. Slonims V. Dockrell J. E. Norbury C. F. (2019). Evidence-based pathways to intervention for children with language disorders. Int. J. Lang. Comm. Disord. 54, 3–19. 10.1111/1460-6984.12387 29696726

[ref9] Goldstein H. (2004). International comparisons of student attainment: some issues arising from the PISA study. Assess. Educ. Princ. Pol. Pract. 11, 319–330. 10.1080/0969594042000304618

[ref10] Hasson N. Camilleri B. Jones C. Smith J. Dodd B. (2013). Discriminating disorder from difference using dynamic assessment with bilingual children. Child Lang. Teach. Ther. 29, 57–75. 10.1177/0265659012459526

[ref11] Hoff E. (2013). Interpreting the early language trajectories of children from low-SES and language minority homes: implications for closing achievement gaps. Dev. Psychol. 49, 4–14. 10.1037/a0027238 22329382 PMC4061698

[ref12] Huang B. H. Chang Y.-H. S. Zhi M. Niu L. (2018). The effect of input on bilingual adolescents’ long-term language outcomes in a foreign language instruction context. Int. J. Biling. 10.1177/1367006918768311

[ref13] Kohnert K. Bates E. (2002). Balancing bilinguals II: lexical comprehension and cognitive processing in children learning Spanish and English. J. Speech Lang. Hear. Res. 45, 347–359. 10.1044/1092-4388(2002/027) 12003516

[ref14] Lugo-Neris M. J. Peña E. D. Bedore L. M. Gillam R. B. (2015). Utility of a language screening measure for predicting risk for language impairment in bilinguals. Am. J. Speech Lang. Pathol. 24, 426–437. 10.1044/2015_AJSLP-14-0061 25885932 PMC4657524

[ref15] Meroni E. C. Vera-Toscano E. Costa P. (2015). Can low skill teachers make good students? Empirical evidence from PIAAC and PISA. J. Policy Model 37, 308–323. 10.1016/j.jpolmod.2015.02.006

[ref16] Neuman S. B. Kaefer T. Pinkham A. M. (2018). A double dose of disadvantage: language experiences for low-income children in home and school. J. Educ. Psychol. 110, 102–118. 10.1037/edu0000201

[ref17] O’Connor M. O’Connor E. Tarasuik J. Gray S. Kvalsvig A. Goldfeld S. (2018). Academic outcomes of multilingual children in Australia. Int. J. Speech Lang. Pathol. 20, 393–405. 10.1080/17549507.2017.1292546 28425775

[ref18] OECD (2014). PISA 2012 results: What students know and can do – student performance in mathematics, reading and science. Vol. 1, Paris: OECD Publishing.

[ref19] OECD (2015). Students, computers and learning. Paris: OECD Publishing.

[ref20] OECD (2016). PISA 2015 results. Vol. I, Paris: OECD Publishing.

[ref21] OECD (2018a). International migration outlook 2018. Paris: OECD Publishing.

[ref22] OECD (2018b). The resilience of students with an immigrant background. Paris: OECD Publishing.

[ref23] Pace A. Alper R. Burchinal M. R. Golinkoff R. M. Hirsh-Pasek K. (2019). Measuring success: within and cross-domain predictors of academic and social trajectories in elementary school. Early Child. Res. Q. 46, 112–125. 10.1016/j.ecresq.2018.04.001

[ref24] PIRLS (2016). What makes a good reader: International findings from PIRLS 2016. Boston: TIMSS & PIRLS International Study Center.

[ref25] Pontoppidan M. Keilow M. Dietrichson J. Solheim O. J. Opheim V. Gustafson S. (2018). Randomised controlled trials in Scandinavian educational research. Educ. Res. 60, 311–335. 10.1080/00131881.2018.1493351

[ref26] Rolstad K. Mahoney K. Glass G. V. (2005). The big picture: a meta-analysis of program effectiveness research on English language learners. Educ. Pol. 19, 572–594. 10.1177/0895904805278067

[ref27] Salameh E.-K. Håkansson G. Nettelbladt U. (1996). The acquisition of Swedish as second language in a group of arabic-speaking pre-school children: word order patterns and phrasal morphology. Logoped. Phoniatr. Vocol. 21, 163–170. 21275588 10.3109/14015439609098885

[ref28] Scheidnes M. Tuller L. (2016). Assessing successive bilinguals in two languages: a longitudinal look at English-speaking children in France. J. Commun. Disord. 64, 45–61. 10.1016/j.jcomdis.2016.10.001 27973321

[ref29] Semel E. Wiig E. H. Secord W. A. (2013). Clinical evaluation of language fundamentals, fourth edition (CELF-4), Swedish version. Stockholm, Sweden: Pearson Assessment.

[ref30] Spencer S. Clegg J. Lowe H. Stackhouse J. (2017). Increasing adolescents’ depth of understanding of cross-curriculum words: an intervention study. Int. J. Lang. Comm. Disord. 52, 652–668. 10.1111/1460-6984.12309 28421646

[ref31] Swedish National Agency for Education (2006). Equity trends in the Swedish school system – a quantitative analysis of variation in student performance and equity from a time perspective. Stockholm: Skolverket.

[ref32] Swedish National Agency for Education (2019). Information on pupil characteristics. Available at: https://www.skolverket.se/skolutveckling/statistik (Accessed April 4, 2019).

[ref33] Thordardottir E. Brandeker M. (2013). The effect of bilingual exposure versus language impairment on nonword repetition and sentence imitation scores. J. Commun. Disord. 46, 1–16. 10.1016/j.jcomdis.2012.08.002 23021785

[ref34] Thordardottir E. Juliusdottir A. G. (2013). Icelandic as a second language: a longitudinal study of language knowledge and processing by school-age children. Int. J. Biling. Educ. Biling. 16, 411–435. 10.1080/13670050.2012.693062

[ref35] TIMSS (2015). TIMSS 2015 international results report. Boston: TIMSS & PIRLS International Study Center.

[ref36] Wang S. Rubie-Davies C. M. Meissel K. (2018). A systematic review of the teacher expectation literature over the past 30 years. Educ. Res. Eval. 24, 124–179. 10.1080/13803611.2018.1548798

[ref37] Windsor J. Kohnert K. (2004). The search for common ground: part I. Lexical performance by linguistically diverse learners. J. Speech Lang. Hear. Res. 47, 877–890. 10.1044/1092-4388(2004/065) 15324292

